# *Rhamnus davurica* leaf extract inhibits Fyn activation by antigen in mast cells for anti-allergic activity

**DOI:** 10.1186/s12906-015-0607-6

**Published:** 2015-03-25

**Authors:** Ji Hyung Kim, A-Ram Kim, Hyuk Soon Kim, Hyun Woo Kim, Young Hwan Park, Jueng Soo You, Yeong Min Park, Erk Her, Hyung Sik Kim, Young Mi Kim, Wahn Soo Choi

**Affiliations:** KU open Innovation Center, School of Medicine, Konkuk University, Chungju, 380-701 Korea; College of Pharmacy, Sungkyunkwan University, Suwon, 440-746 Korea; College of Pharmacy, Duksung Women’s University, Seoul, 132-714 Korea

**Keywords:** *Rhamnus davurica*, Herbal medicine, Mast cells, Allergy, Anaphylaxis, Fyn

## Abstract

**Background:**

Complementary and alternative herbal medicines are recently considered as a promising approach for treating various diseases. We screened approximately 100 plant extracts for anti-allergic activity. *Rhamnus davurica* leaf extract showed the most potent inhibitory effect on the activation of RBL-2H3 mast cells. Although *Rhamnus davurica* extract has been used to treat pruritus, dysuresia, and constipation as a traditional herbal medicine in some Asian countries, an anti-allergic effect of *Rhamnus davurica* has not yet been demonstrated. We aimed to investigate the effect and mechanism of the leaf extract of *Rhamnus davurica* (LERD) on mast cells in vitro and allergic responses in vivo.

**Methods:**

The effects of LERD on the activation of mast cells and mast cell-mediated passive cutaneous anaphylaxis (PCA) were measured in mice and two types of mast cells, mouse bone marrow-derived mast cells (BMMCs) and RBL-2H3 cells in vitro. A mechanistic study of its inhibitory effect was performed by using degranulation assay, reverse transcriptase-polymerase chain reaction, enzyme-linked immunosorbent assay, and western blotting analysis.

**Results:**

LERD reversibly suppressed antigen-stimulated degranulation in BMMCs and RBL-2H3 cells, and also inhibited mRNA expression and secretion of TNF-α and IL-4 in a dose-dependent manner. In a PCA animal model, LERD significantly inhibited antigen-induced allergic response and degranulation of ear tissue mast cells. As for the mechanism of action, LERD inhibited the activation of Syk, which is the pivotal signaling protein for mast cell activation by antigen. Furthermore, LERD also impeded the activations of well-known downstream proteins such as LAT, Akt and three MAP kinases (Erk, p38 and JNK). In an in vitro kinase assay, LERD suppressed the activation of Fyn in antigen-stimulated mast cells.

**Conclusion:**

This study demonstrated for the first time that LERD has anti-allergic effects through inhibiting the Fyn/Syk pathway in mast cells. Therefore, this study provides scientific evidence for LERD to be used as an herbal medicine or health food for patients with allergic diseases.

## Background

Allergic diseases such as allergic asthma, allergic rhinitis, and atopic dermatitis have been increasing, particularly in many developed countries. Generally, around 10 to 20% of the population are reported as presenting with allergic diseases in those countries [1,2]. Usually, patients with allergy-prone genetic factors have type 2 helper T cells (Th2) reactions under a certain environment. When patients are exposed to potential allergens such as milk, egg, nuts, and shellfish, various allergic symptoms may occur. In the course of early and late allergic reactions, mast cells are solidly acknowledged as one of the major culprit effector cells [3].

Mast cells play a key role in prompting a variety of allergic symptoms [4,5]. Mast cells have FcεRI, a high-affinity receptor for IgE, which makes a complex with antigen-specific IgE. When mast cells are stimulated by an antigen, various allergic mediators including histamines, eicosanoids, and pro-inflammatory cytokines are secreted from mast cells [6,7]. Thus, mast cells play a critical role in causing allergic diseases, and therefore mast cells-stabilizing therapies have been increasingly reported [8,9].

When mast cells are stimulated by antigens, the initial activation of Lyn or other Src-family kinases is observed for the phosphorylation of an immunoreceptor tyrosine activation motif (ITAM) of FcεRI γ subunit and Syk is, then, recruited to tyrosine phosphorylated ITAMs for the full activation of Syk [10,11]. This event leads to the activation of downstream signaling molecules including a linker for the activation of T cells (LAT), SLP-76, Gab2, phospholipase (PL) Cγ, and MAP kinases. This series of activations of signaling proteins leads to the full activation of mast cells in prompting allergic responses [12,13].

The Src family kinases, including Lyn, Fyn, and Fgr, are activated by antigen stimulation in mast cells [11]. The initial activation of Lyn is essential for the activation of mast cells, and Lyn additionally also has a negative role in mitigating the excess activation of mast cells [14]. Whereas Fyn and Fgr show solely a positive role in the activation of Syk and its downstream signaling cascades for mast cell activation [11]. Therefore, a Src-family kinase-targeted study could be an efficient direction for treatment of allergic diseases.

*Rhamnus davurica* is widely found throughout fields of Korea, China, and other Asian countries. Extract of *Rhamnus davurica* has long been in use as a folk remedy in the treatment of several diseases including pruritus, dysuresia, and constipation. However, the effect of *Rhamnus davurica* on allergic diseases remains to be unclear.

In this study, we investigated the anti-allergic effects of the leaf extract of *Rhamnus davurica* (LERD) in mast cells cultures and in passive cutaneous anaphylaxis animal models. LERD suppressed the activation of mast cells and anaphylaxis responses through the inhibition of the activation of Fyn/Syk pathway in antigen-stimulated mast cells.

## Methods

### Reagents

Antibodies that work against the phosphorylated forms of Akt, Erk1/2, p38, JNK, Syk (Y525/526), and LAT (Y191) were obtained from Cell Signaling Technology, Inc. (Danvers, MA, USA). The 4-Amino-5-(4-chlorophenyl)-7-(dimethylethyl)pyrazolo [3,4-*d*]pyrimidine (PP2) was obtained from Calbiochem (La Jolla, CA, USA). Enzyme-linked immunosorbant assay (ELISA) kits for analyzing IL-4 in media was obtained from Invitrogen-Biosource Cytokine & Signaling (Camarillo, CA, USA). An ELISA kit for TNF-α measurement was from R&D Systems, Inc. (Minneapolis, MN, USA). Reagents for cell culture media were obtained from GIBCO/Life Technologies, Inc. (Rockville, MD, USA). Most of all the other reagents used were purchased from Sigma-Aldrich (St. Louis, MO, USA).

### Animals

BALB/c mice (5-weeks old) were used for the isolation of bone marrow-derived mast cells (BMMCs) and the induction of passive cutaneous anaphylaxis (PCA). All animal studies were performed according to institutional guidelines after obtaining approval from the Institutional Animal Care and Use Committee (IACUC) at Konkuk University.

### Mast cell preparation and cell culture

BMMCs were isolated from the thigh bones of 5 week-old BALB/c mice as in previous studies [15]. The BMMCs were cultured in media (RPMI 1640), containing 2 mM L-glutamine, 0.1 mM nonessential amino acids, antibiotics, and 10% fetal bovine serum (FBS) containing 10 ng/ml IL-3 in 5% CO_2_, 37°C incubator. Four weeks following isolation, the BMMCs were used for the following experiments. The RBL-2H3 cells from ATCC (American Type Culture Collection, VA, USA) were cultured in a minimum essential medium (MEM) with Earle’s salts, supplemented with glutamine, antibiotics, and 15% FBS.

### Preparation of LERD and other plant extracts

Leaf of *Rhamnus davurica* was collected from Hantaek Botanical Garden (Yongin-si, Korea) and was authenticated by the Plant Extract Bank at the Korea Research Institute of Bioscience and Biotechnology (Daejeon, Korea). The methanol extracts of *Rhamnus davurica* leaf (LERD) and other plants were manufactured according to the institute’s standard protocol. The yield of the extraction process was approximately 15% of total dry leaf amount. The extracted and plant specimen (017-005 for LERD or as indicated in Table 1) were deposited at the Plant Extract Bank and Konkuk University. The extracts were solubilized in dimethyl sulfoxide (DMSO) for cell culture experiments and suspended in 5% Gum arabic for oral administration of extracts in the animal study.

**Table 1:** **Effects of plant extracts on the Ag-induced degranulation in RBL-2H3 mast cells**

**Plant name**	**Part extracted**	**Voucher specimen number**	**Percent inhibition of degranulation** ^**a**^
*Acer okamotoanum*	Stem-bark	016-081	0.00
*Acer ukurunduense*	Leaf	016-050	40.78
*Acer ukurunduense*	Stem	016-051	30.32
*ActinoStemma lobatum*	Whole plant	017-038	18.48
*Ardisia pusilla*	Whole plant	016-061	0.00
*Aster incisa*	Whole plant	016-001	0.00
*Berberis amurensis var. quelpaertensis*	Stem	016-069	16.96
*Betula chinensis*	Leaf	017-013	39.17
*Betula chinensis*	Stem	017-014	27.76
*Betula davurica*	Stem	017-045	3.78
*Betula davurica*	Stem-bark	017-046	0.00
*Betula ermani var. saitoana*	Leaf	016-089	10.80
*Betula ermani var. saitoana*	Stem	016-090	1.78
*Boehmeria pannosa*	Whole plant	016-080	0.43
*Bupleurum longeradiatum*	Whole plant	017-016	29.22
*Cacalia auriculata var. matsumurana*	Whole plant	016-047	12.05
*Campylotropis macrocarpa*	Aboveground part	017-023	0.00
*Celosia argentea*	Whole plant	017-012	38.14
*Chaenomeles sinensis*	Leaf	017-040	3.85
*Cinnamomum loureirii*	Leaf	016-082	5.92
*Clerodendrum trichotomum*	Leaf	016-026	2.27
*Clintonia udensis*	Whole plant	016-019	51.37
*Corydalis heterocarpa*	Whole plant	016-079	11.79
*Crataegus scabrida*	fruit	017-047	0.00
*Cyrtomium falcatum*	Aboveground part	016-028	4.84
*Daphniphyllum macropodum*	Leaf	017-019	6.46
*Daphniphyllum macropodum*	Stem	017-020	0.29
*Deutzia prunifolia*	Stem	017-051	44.69
*Dicentra spectabilis*	Aboveground part	016-010	8.22
*Diospyros kaki*	Leaf	017-007	17.98
*Diospyros kaki*	Stem	017-008	17.68
*Diospyros kaki*	Stem-bark	017-009	17.25
*Diospyros kaki*	Root	017-011	11.64
*Dryopteris championi*	Whole plant	016-088	6.97
*Elaeagnus macrophylla*	Leaf	017-059	23.52
*Forsythia nakaii*	Leaf	016-085	1.93
*Fraxinus sieboldiana*	Leaf	017-073	9.30
*Geranium wilfordii*	Whole plant	017-070	42.07
*Houttuynia cordata*	Whole plant	016-077	7.45
*Hydrangea serrata for. acuminata*	Aboveground part	016-058	67.16
*Hypericum erectum*	Whole plant	017-018	6.53
*Kalopanax pictus*	Stem-heartwood	016-083	14.84
*Lilium lancifolium*	Aboveground part	016-091	8.82
*Lindera erythrocarpa*	Leaf	016-054	18.09
*Lindera erythrocarpa*	Stem	016-055	14.20
*Lindera obtusiloba*	Stem	016-063	19.80
*Lycopodium clavatum var. nipponicum*	Whole plant	017-069	23.56
*Lycoris aurea*	Underground part	017-015	52.03
*Melandryum seoulense*	Whole plant	017-003	62.76
*Monochoria vaginalis var. plantaginea*	Whole plant	017-043	2.84
*Mosla punctulata*	Whole plant	017-037	6.83
*Nandina domestica*	Leaf	016-023	6.46
*Nuphar japonicum*	Whole plant	016-006	9.22
*Oenothera laciniata*	Whole plant	016-075	14.50
*Pedicularis resupinata*	Whole plant	017-072	35.15
*Perilla frutescens var. acuta*	Whole plant	017-071	14.14
*Persicaria fauriei*	Whole plant	018-003	28.89
*Persicaria thunbergii*	Whole plant	017-017	17.79
*Phlomis koraiensis*	Aboveground part	017-064	19.72
*Phlomis koraiensis*	Underground part	017-065	1.94
*Phtheirospermum japonicum*	Whole plant	017-024	27.81
*Picris hieracioides var. glabrescens*	Whole plant	017-074	16.13
*Polystichum polyblepharum*	Whole plant	016-020	0.99
*Potentilla dickinsii*	Whole plant	017-036	7.18
*Prunus sargentii*	Leaf	017-062	24.50
*Prunus takesimensis*	Stem-bark	016-070	52.74
*Pyrus pyrifolia*	Leaf	017-034	3.85
*Pyrus pyrifolia*	Stem-bark	017-035	11.10
*Rhamnus davurica*	Leaf	017-005	92.35
*Rhododendron tschonoskii*	Leaf	016-099	9.43
*Rhododendron tschonoskii*	Stem	016-100	25.70
*Rhododendron yedoense var. poukhanense*	Stem	016-060	16.80
*Rhodotypos scandens*	Leaf	017-056	14.20
*Rodgersia tabularis*	Whole plant	016-005	3.83
*Salix hallaisanensis*	Stem	016-036	31.12
*Sanguisorba hakusanensis*	Whole plant	017-068	10.22
*Sanguisorba tenuifolia var. alba*	Whole plant	017-002	29.37
*Sorbus commixta*	Stem-bark	016-037	22.84
*Sorbus commixta*	Leaf	016-038	10.86
*Sorbus commixta*	Stem	016-039	14.46
*Sorbus commixta*	fruit	017-039	10.32
*Spiraea salicifolia*	Leaf	016-016	0.00
*Stipa sibirica*	Whole plant	017-025	46.66
*Suaeda asparagoides*	Whole plant	017-026	27.48
*Symplocos coreana*	Stem	016-067	3.90
*Syringa velutina*	Leaf	016-093	0.55
*Syringa velutina*	Stem	016-094	0.35
*Tilia insularis*	Stem-bark	016-071	10.69
*Tripterygium regelii*	Stem	017-048	31.59
*Ulmus laciniata*	Stem-bark	016-022	0.00
*Veronica kiusiana*	Whole plant	017-028	3.07
*Veronica longifolia*	Aboveground part	017-022	4.14
*Viburnum sargentii*	Leaf	017-052	4.31
*Vicia amoena*	Whole plant	017-006	27.44
*Vicia angustifolia var. minor*	Whole plant	017-001	17.50
*Vicia unijuga*	Whole plant	017-027	28.89
*Vitex rotundifolia*	Leaf	017-075	0.63
*Vitis thunbergii var. sinuata*	Whole plant	016-014	0.11
*Zizyphus jujuba*	fruit	017-042	0.00

^a^The degranulation was assessed through the measurement of the release of the granule marker β-hexosaminidase from RBL-2H3 mast cells as described in the “Methods” section. The percent inhibition of degranulation is presented as the mean values from three independent experiments.

### Degranulation assay in mast cells

Mast cells (1.8 × 10^5^/well) were primed in 50 ng/ml anti-dinitrophenol (DNP) IgE on 24-well plates for 12 h. The cells were then washed twice with 1,4-piperazinediethanesulfonic acid (PIPES)-buffered medium [25 mM PIPES (pH 7.2), 159 mM NaCl, 5 mM KCl, 0.4 mM MgCl_2_, 1 mM CaCl_2_, 5.6 mM glucose, and 0.1% fatty acid-free fraction V from bovine serum] for RBL-2H3 cells or with Tyrode buffer [20 mM HEPES (pH 7.4), 135 mM NaCl, 5 mM KCl, 1.8 mM CaCl_2_, 1 mM MgCl_2_, 5.6 mM glucose, and 0.05% bovine serum albumin (BSA)] for BMMCs and then pre-incubated in the buffer for 1 h with or without each plant extract. The mast cells were stimulated by the antigen (DNP-BSA) for 10 min and the stimulation was terminated using ice. The cultured media were transferred to new tubes and cells were disrupted with 0.1% triton X-100. For β-hexosaminidase assay, the culture media and cell lysates were mixed with 1 mM *p*-nitrophenyl-N-acetyl-β-D-glucosaminide on 96 well plates and incubated at 37°C for 1 h. Next, 0.1 M carbonate was used to stop the reaction. The density of the color was measured at 405 nm by a microplate reader. Degranulation of mast cells was determined by calculating the ratio of β-hexosaminidase activity released into the culture medium to the total activity of β-hexosaminidase from the cell lysate plus the culture medium [16].

### Assay of cell viability

BMMCs (5 × 10^4^/well) were plated on 96-well plates in serum-free RPMI-1640 medium with or without LERD for 8 h. Then, cell viability was determined by using a cell counting kit-8 (CCK-8) (Dojindo Laboratories, Kumamoto, Japan), according to the manufacturer's protocol. CCK-8 solution was added to each well of the plate at 1:10 ratio to volume of medium, and the plates were incubated for 1 h in a CO_2_ incubator at 37°C. The absorbance of color density was measured at 450 nm.

### Measurement of TNF-α and IL-4 expression by reverse transcriptase-polymerase chain reaction (RT-PCR)

To obtain the total RNA from cells, an Easy-spinTM Total RNA Extraction Kit (iNtRON Biotechnology, Inc., Sungnam, Korea) was used. PCR was performed at 94°C for 20 sec, at 62°C for 10 sec, and at 72°C for 20 sec for 30 cycles. The primers were used as follows: rat TNF-α forward 5′-ACCACGCTCTTCTGTCTACTGAAC-3′; rat TNF-α reverse: 5′-CCGGACTCCGTGATGTCTAAGTACT-3′; rat IL-4 forward 5′-ACCTTGCTGTCACCCTGTTC-3′; rat IL-4 reverse 5′-TTGTGAGCGT GGACTCATTC-3′; rat glyceraldehyde-3-phosphate dehydrogenase (GAPDH) forward 5′-GTGGAGTCTACTGGCGTCTTC-3′; rat GAPDH reverse: 5′-CCAAGGC TGTGGGC AAGGTCA-3′.

### Measurement of TNF-α and IL-4 in culture media by ELISA

RBL-2H3 cells (5.0 × 10^5^/well/12 well-clustered plate) were plated with 50 ng/ml DNP-specific IgE overnight. Cells were stimulated by antigen with or without LERD for 8 h at 37°C and then the culture media were analyzed via the ELISA kit according to manufacturer’s protocol.

### Immunoblotting analysis

The IgE-primed RBL-2H3 cells were stimulated with 25 ng/ml antigen for 7 min or as indicated. The cells were lysed with ice-cold lysis buffer (20 mM HEPES, pH 7.5, 150 mM NaCl, 1% Nonidet p-40, 10% glycerol, 60 mM octyl β-glucoside, 10 mM NaF, 1 mM Na_3_VO_4_, 1 mM phenylmethylsulfonyl fluoride, 2.5 mM nitrophenylphosphate, 0.7 μg/ml pepstatin and protease inhibitor cocktail tablet). Lysates were kept on ice for 30 min and then centrifuged at 13,000 × *g* for 10 min at 4°C. After centrifuging, the supernatant proteins were denatured at 95°C for 5 min in a 3× Laemmli buffer [17]. The denatured proteins were separated by sodium dodecylsulfate (SDS)-polyacrylamide gel electrophoresis (PAGE) and then transferred to a nitrocellulose membrane. The transferred protein membrane was blocked in tris-buffered saline-0.05% Tween 20 (TBS-T) buffer containing 5% BSA. The membrane was incubated overnight with the specific antibody for the target protein. After washing the membrane with TBS-T buffer, it was incubated with a labeled secondary antibody directed against the primary antibody. The protein bands for immunoreactive proteins were detected with horseradish peroxidase (HRP)-coupled secondary antibodies and enhanced chemiluminescence according to manufacturer’s protocol (Amersham Biosciences, Piscataway, NJ, USA).

### Passive cutaneous anaphylaxis (PCA) and histological analysis

PCA was generated in mice according to a previous study [18]. DNP-specific IgE (0.5 μg per mouse) was intradermally injected into the right ear of a BALB/c mouse (male, 5 weeks old). After 24 h, LERD (0, 100, 300, and 1,000 mg/kg) or cetirizine (20 mg/kg) was orally administered to the mice. After 60 min, the mice were intravenously injected with 250 μg of antigen in Evans blue solution (5 mg/ml PBS). The ears were collected after the mice were euthanized 1 h later. The dye of ear tissue was extracted overnight in 700 μl of formamide at 63°C and then the absorbance was analyzed at 620 nm. For histological analysis, ear tissues were fixed in 4% paraformaldehyde in PBS for 24 h. 5-μm paraffin sections were stained with 0.1% toluidine blue and examined with an optical microscope (Olympus DP 70, Center Valley, PA, USA) at × 100 magnification. Degranulated mast cells in the ear tissue were counted as previously described [19].

### Measurement of tyrosine kinase activity in vitro

After stimulating IgE-primed mast cells with 25 ng/ml antigen for 7 min, Lyn or Fyn were immunoprecipitated from whole cell lysates by using specific antibodies. The activity of tyrosine kinase was measured using a Universal Tyrosine Kinase Assay Kit (Gen Way, San Diego, USA) according to manufacturer’s instruction.

### Presentation of results

The data were presented as the means ± SEM from three or more independent experiments. Statistical analysis was performed by using one-way ANOVA and the Dunnett test. All statistical calculations (*P < 0.05 and **P < 0.01) were performed with SigmaStat software (Systat Software Inc., Point Richmond, CA, USA).

## Results

### The effects of LERD and other herbal extracts on antigen-stimulated degranulation in mast cells

**Figure 1:**
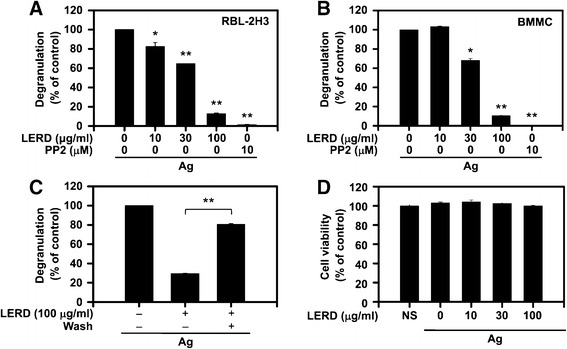
**LERD reversibly inhibits antigen-stimulated degranulation in mast cells.** RBL-2H3 mast cells **(A)** and BMMCs **(B)** were incubated overnight in 24-well cluster plates with 50 ng/ml of IgE in a complete growth medium. The IgE-primed mast cells were stimulated by antigen (25 ng/ml) with or without LERD. The activity of β-hexosaminidase was measured as described in the “Methods” section. **(C)** RBL-2H3 cells were pre-incubated for 1 h with 0.1% DMSO or 100 μg/ml LERD. After LERD-treated cells were washed five times with PIPES buffer, degranulation of mast cells was measured as for panel **A**. **(D)** BMMCs were incubated with LERD for 8 h and the cell viability, then, was measured as described in the “Methods” section. The mean ± SEM from three independent experiments are shown here. Asterisks indicate significant differences from the controls, *P < 0.05 and **P < 0.01. PP2 is a general Src family kinase inhibitor.

Mast cells have secretary granules containing allergic mediators such as histamine and various proteases that cause allergic symptoms [20]. Thus, the effects of approximately 100 herbal extracts on the degranulation of mast cells were firstly measured in RBL-2H3 mast cells. The extracts from *Vitex rotundifolia, Prunus sargentii, Lycoris aurea, Hydrangea serrata for. acuminata*, *Prunus takesimensis*, *Clintonia udensis*, and *Rhamnus davurica* significantly inhibited degranulation (>50%) at a concentration of 100 μg/ml in RBL-2H3 cells (Table 1). Among them, LERD most potently suppressed degranulation, release of β-hexosaminidase, in antigen-stimulated RBL-2H3 mast cells or BMMCs in a dose dependent manner (Figure 1A and B). However, LERD did not inhibit the activity of β-hexosaminidase released from mast cells (data not shown). When mast cells were washed twice with the incubation buffer after pretreating with LERD for 1 h, the degranulation of mast cells was almost completely recovered (Figure 1C), indicating that the effect of LERD on mast cell activation was reversible. Notably, no effect on the viability of mast cells was observed by LERD at the experimental doses (Figure 1D).

### Effect of LERD on the expression and secretion of inflammatory cytokines

**Figure 2:**
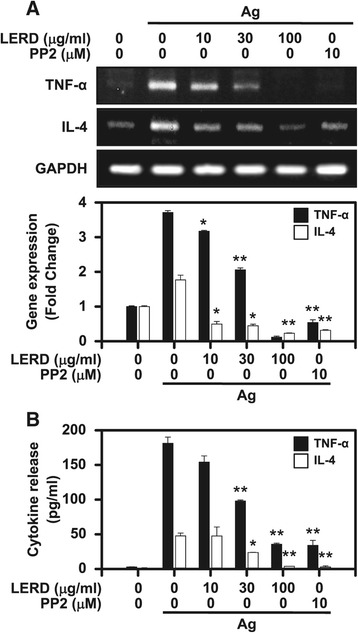
**The effect of LERD on the expression and secretion of TNF-α and IL-4. (A)** The IgE-primed RBL-2H3 cells (1 × 10^6^ cells/well) were stimulated by the antigen for 15 min for the assay of TNF-α and IL-4 mRNA by RT-PCR. The representative images (upper panel) and densitometric data (lower panel) from three independent experiments are shown. **(B)** The IgE-primed RBL-2H3 cells (1 × 10^6^ cells/well) were stimulated by antigen for 8 h and the secretion of TNF-α and IL-4 was then measured by ELISA. The values are expressed as the mean ± SEM from three independent experiments. Asterisks indicate significant differences from the controls, *P < 0.05 and **P < 0.01. PP2 is a general Src family kinase inhibitor.

In addition to degranulation in mast cells, mast cells also secrete various inflammatory cytokines. Among them, TNF-α and IL-4 are secreted in antigen- stimulated cells. Therefore, we measured the expression levels of TNF-α and IL-4 by RT-PCR. As shown in Figure 2A, the expression of TNF-α and IL-4 was dose-dependently suppressed in antigen-stimulated mast cells. The secretion levels of TNF-α and IL-4 were also analyzed by ELISA assay. The secretion of TNF-α and IL-4 was consistently inhibited in a dose dependent manner (Figure 2B).

### Effect of LERD on activations of signaling molecules by antigen

**Figure 3:**
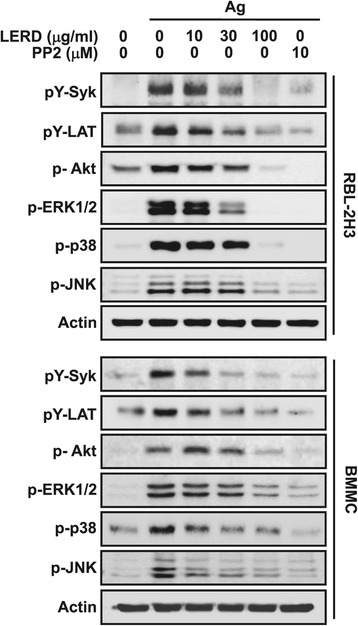
**LERD inhibits the activation of Syk and Syk-mediated downstream signaling molecules.** The RBL-2H3 cells (upper panel) or BMMCs (lower panel) were incubated overnight with 50 ng/ml IgE and cells were then stimulated with 25 ng/ml antigen with or without LERD for 7 min. Cell lysates were subjected to immunoblot analysis with specific antibodies to detect phosphorylated or total proteins. Representative immunoblotting images are shown from three independent experiments. PP2 is a general Src family kinase inhibitor.

Next, to ascertain the mechanism of the LERD action, we investigated which intracellular signaling molecules were affected by LERD treatment. It is well known that Syk and its direct substrate protein LAT pathway is a pivotal signaling cascade for mast cell activation [12]. LERD suppressed the activation of Syk and LAT in RBL-2H3 cells and BMMCs (Figure 3). At a dose of 30 μg/ml, the inhibition was obvious and was nearly complete at 100 μg/ml (Figure 3). Next, we also studied MAP kinase signaling pathway which is generally accepted that three typical MAP kinases are critical for the production of inflammatory cytokines from mast cells [21,22]. In this experiment, LERD significantly reduced the activation of ERK1/2, p-38 and JNK in a dose dependent manner (Figure 3).

### LERD inhibited activity of Fyn, but not Lyn, in antigen-stimulated mast cells

**Figure 4:**
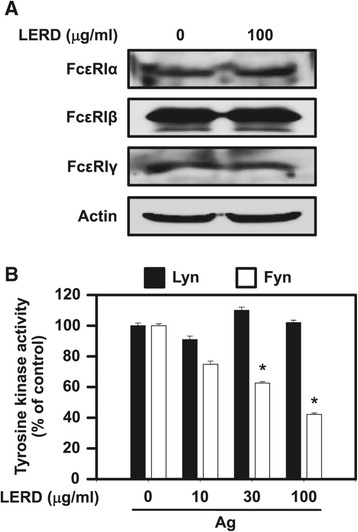
**LERD inhibits the activity of Fyn, but not Lyn.** RBL-2H3 cells (1 × 10^7^ cells/dish) were incubated with 50 ng/ml IgE for overnight and then stimulated with 25 ng/ml antigen for 7 min after pre-incubating with or without LERD for 1 h. **(A)** Cell lysates were subjected to immunoblot analysis with specific antibodies to detect each FcεRI subunit. Representative immunoblotting images are shown from three independent experiments. **(B)** Fyn or Lyn was immunoprecipitated from the cell lysates and the immunoprecipitates were incubated in the kinase assay buffer at room temperature for 40 min and the activity was measured as described in the “Methods” section. The values are expressed as the mean ± SEM from three independent experiments. Asterisks indicate significant differences from the controls, *P < 0.05 and **P < 0.01. PP2 is a general Src family kinase inhibitor.

Several lines of evidence suggest that Lyn is the initially activated tyrosine kinase in antigen-stimulated mast cells. The aggregation of IgE-high affinity receptor (FcεRI) by antigen initially leads to the activation of Lyn for downstream signaling molecules [12]. Another tyrosine kinase, Fyn, is also activated in mast cells by antigen for a complementary pathway to stimulate full activation of mast cells [23]. LERD did not show any suppressive effect on the expressions of FcεRI subunits (Figure 4A). Next, we tested whether LERD suppressed the upstream tyrosine kinases of Syk, Lyn, or Fyn in the cells. As shown in Figure 4B, LERD inhibited activation of Fyn, but not Lyn, in a dose dependent manner.

### Effect of LERD on passive cutaneous anaphylaxis (PCA) in mice

**Figure 5:**
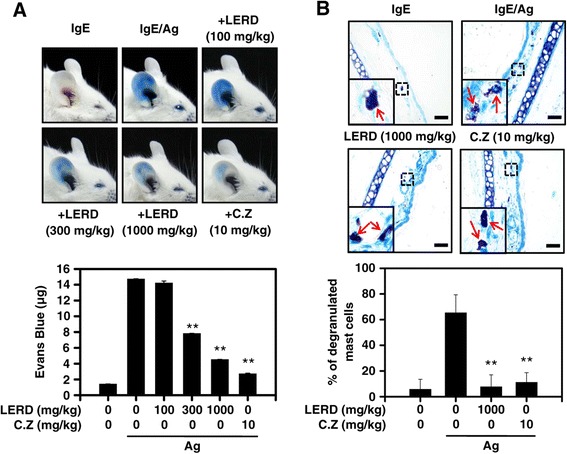
**LERD suppresses passive cutaneous anaphylaxis in vivo.** An IgE (0.5 μg) was intradermally injected into the mouse ear. After 24 h, an injection of 250 μg antigen in 4% Evans blue PBS solution was administered into the mouse tail vein. The LERD was orally administered 1 h before the treatment of the antigen. The mouse was euthanized 1 h after the antigen challenge, and the ear was then removed for measurement of the amount of dye extravasated **(A)** or histological changes **(B)** by antigen treatment. **(A)** Representative photographs of ears after PCA (upper panel) and quantitative data for ear-tissue content of Evans blue (lower panel). **(B)** The ear tissues were prepared and stained with toluidin blue for histological examination as described in the “Methods” section. Representative images (upper panel) and the percentage of degranulated mast cells in ear skin sections (lower panel) are shown: arrows indicate mast cells. bar, 50 μm. The values are expressed as the mean ± SEM from three independent experiments. *P < 0.05 and **P < 0.01.; C.Z., cetirizine.

To determine the anti-allergic effect of LERD in vivo, a PCA model was used in BALB/c mice. Histological analysis was further performed to check whether LERD inhibited mast cell degranulation in mouse ear tissues. The PCA responses were successfully induced by the injection of IgE/antigen in mice. LERD significantly inhibited the response in a dose dependent manner (Figure 5A). In the histological analysis with ear tissues, LERD suppressed the degranulation of tissue mast cells by antigen (Figure 5B). These results suggested that LERD has an anti-allergic effect in vivo through the inhibition of mast cells.

## Discussion

Allergic diseases such as asthma, allergic rhinitis, and atopic dermatitis are on the rise, particularly, in developed countries. It has been generally accepted that when people who are skewed to Th2 intake or come into physical contact with a specific allergen, they suffer from allergic symptoms. A large body of evidence suggests that mast cell is a key player of acute and chronic allergic inflammation in allergic diseases [20]. In this context, stimulation of mast cells by antigen results in the release of a variety of mediators such as histamine and an array of inflammatory cytokines that cause allergic symptoms [6,12]. Therefore, the regulation of mast cells is one of the potential therapeutic approaches for the development of allergy medication. In this study, we demonstrated for the first time that LERD suppresses mast cell-mediated PCA in mice by inhibiting the activation of the Fyn/Syk signaling pathway in mast cells.

Mast cells play a critical role in immune response when a foreign antigen infiltrates a human body. When mast cells are stimulated by antigen, they release preformed granule-associated mediators, such as histamine, serotonin, and β-hexosaminidase, eicosanoids, inflammatory cytokines, and chemokines [24,25]. These mediators lead to certain pathophysiological changes and tissue remodeling for various allergic symptoms.

The effects of drugs are generally reversible in a human body. After a certain period following the administration of a drug, the function of drug-target molecule returns to normal functioning status. If the action of a drug, on the other hand, is irreversible, its effect persists until the body generates additional protein. Therefore, it is critical to check at the early stage of the drug development whether a candidate is reversible. The inhibition of LERD on mast cell activation was reversible (Figure 1C), suggesting that LERD achieves its effect by reversibly suppressing the activations of signaling molecules in antigen-stimulated mast cells.

Accumulating evidence from mice and humans has argued that Th2 cytokines such as IL-4, IL-5, and IL-13 are contributors to allergic responses [26]. Mast cells secrete the cytokines. Apart from these Th2 cytokines, TNF-α, one of the critical inflammatory cytokines, is also secreted from mast cells [12]. In this study, LERD suppressed the expression and secretion of IL-4 and TNF-α in antigen-stimulated mast cells (Figure 2). The results further suggested that LERD may have a therapeutic potential in cytokine-associated allergic symptoms in allergic patients.

In the atopic environment, mast cells are activated by binding antigen to the IgE that is bound to its multimeric receptor, FcɛRI. The aggregation of FcɛRIs leads to the phosphorylation of tyrosine residues of the ITAMs of FcɛRI by Lyn and, subsequently, activation of Syk, a pivotal signaling molecule for mast cell activation by the antigen. Syk is essential for the activation of many downstream signaling molecules including LAT adaptor protein. The activation of Syk is also critical for the activation of Akt and three typical MAP kinases (ERK1/2, p38, and JNK). In such a manner, mast cells are activated to release an array of allergic mediators [12]. In this study, LERD inhibited activation of Syk and LAT in antigen-stimulated mast cells (Figure 3), suggesting that its inhibition of Syk activation was a key mechanism of LERD.

Fyn is an essential signaling molecule for full activation of antigen-stimulated mast cells. The activation of Fyn is critical for the activation of the Gab2/PI3-K/Akt pathway [23]. In other reports, Fyn is also important for the activation of Syk [11,27]. Of note, our study found that LERD inhibited only the activity of Fyn, not Lyn, in antigen-stimulated mast cells (Figure 4B), suggesting that LERD suppressed the activation of Fyn and Fyn/Syk-mediated downstream signaling molecules.

Anaphylaxis is an allergic response that is remarkably dangerous and acute as it can cause death due to asphyxiation. Systemic or local anaphylaxis may occur when the exterior allergens such as insect venom, food, and pollen infiltrate the allergic patient’s body [28]. The reactions cause the release of inflammatory mediators and cytokines from mast cells and basophils [29]. To confirm the anti-allergic effect of LERD in animal model, we utilized IgE-mediated PCA mice. In the mice, LERD remarkably suppressed the anaphylactic responses (Figure 5A) and degranulation of mast cells in ear (Figure 5B). Taken together, the results demonstrated that LERD has an anti-allergic effect in vivo.

## Conclusions

**Figure 6:**
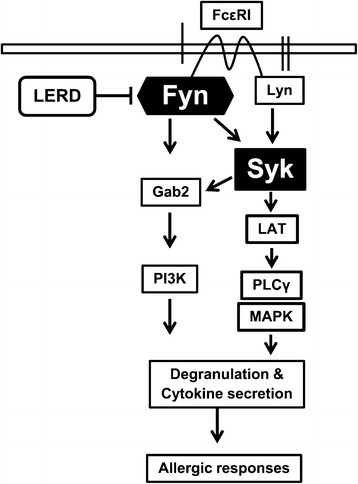
**LERD inhibits the activation of Fyn for the antigen-mediated activation of mast cells.** LERD suppresses the activation of Fyn and Fyn/Syk-mediated downstream signaling molecules for the release of various allergic mediators in antigen-stimulated mast cells.

Our results demonstrated for the first time that LERD suppresses degranulation and cytokine production in antigen-stimulated mast cells and, furthermore, inhibits IgE-mediated allergic responses in mice. Mechanically, LERD suppresses the activation of Fyn/Syk pathway and its downstream signaling molecules to supress secretion of allergic mediators in mast cells (Figure 6). Although further comprehensive studies for bioavailability, toxicity, and active components of LERD are required for the development of drug, our finding suggested that LERD may be a useful herbal extract for human allergic diseases.

## Abbreviations

BMMC: Bone marrow–derived mast cell
DNP: 2,4-Dinitrophenyl
LAT: Linker for activation of T cells
MAP: Mitogen-activated protein
PCA: Passive cutaneous anaphylaxis
